# The dog erythrocyte antigen 1 blood group in nondomesticated canids and compatibility testing between domestic dog and nondomesticated canid blood

**DOI:** 10.1111/jvim.15950

**Published:** 2020-11-03

**Authors:** Thomas Charpentier, Thierry Petit, Maryline Guidetti, Isabelle Goy‐Thollot

**Affiliations:** ^1^ Zoo de La Palmyre Les Mathes France; ^2^ Dianov Laboratories Limonest France; ^3^ VetAgro Sup – SIAMU Marcy l'Etoile France

**Keywords:** blood compatibility, *Canidae*, dog erythrocyte antigen, nondomesticated canids, red cell antigens

## Abstract

**Background:**

The dog erythrocyte antigen (DEA) 1 blood group is considered as the most immunogenic and clinically important in dogs. Little is known in nondomesticated canids.

**Objectives:**

To type DEA 1 in nondomesticated captive canids and to evaluate potential interspecific blood transfusions between domestic and nondomestic canids.

**Animals:**

One hundred forty captive nondomesticated canids belonging to 13 species from 19 French zoos, and 63 domestic dogs.

**Methods:**

Prospective study. Blood samples were typed for DEA 1 using immunochromatographic and flow cytometric techniques. A neutral gel column test was used for crossmatching.

**Results:**

Of 140 nondomesticated canids, 72.9% were DEA 1+ and 27.1% were DEA 1− using immunochromatographic technique and 74.3% were DEA 1+ and 25.7% were DEA 1− by flow cytometric technique.

Crossmatch (XM) between 140 nondomesticated canid red blood cells (RBCs) and plasma from a previously DEA 1+ sensitized DEA 1− dog revealed 112 incompatibilities (80%). Crossmatches between 130 nondomesticated canid serum and 1 or up to 8 donor dogs' RBCs revealed 99 of 130 (76%) compatibilities. Crossmatches between 115 nondomesticated canid RBCs and donor dogs' serum revealed 59 of 115 (51%) compatibilities.

**Conclusions and Clinical Importance:**

Dog erythrocyte antigen 1 blood type is present in nondomesticated canids with variable prevalence depending on species. The majority of tested nondomesticated canids appear to have no naturally occurring alloantibodies against domestic dogs' RBCs. Therefore xenotransfusion of blood from domestic dogs can be considered when species specific blood is not available. Cross matching is essential before xenotransfusion.

**Abbreviations**
(p)RBC(s): (packed) red blood cell(s)
DEA: dog erythrocyte antigen
EDTA: ethylenediaminetetraacetic acid
GC: gel column
MFI: mean fluorescence intensity
PBS: phosphate buffered saline
SD: sensitized dog
XM: crossmatch

## 1 INTRODUCTION

Seven canine blood groups are internationally recognized and have been classified as dog erythrocyte antigens (DEA): DEA 1, 3, 4, 5, 6, 7, and 8. More recently, some new groups have been described: Dal, Kai 1.1, and Kai 1.2.^1^, ^2^ The DEA 1 is considered clinically as the most important blood group in dogs because of its strong antigenicity and nearly equal distribution of DEA 1+ and DEA 1− dogs among many breeds worldwide. Distribution of DEA 1+ and DEA 1− dogs could depend on the breed.^2^, ^3^, ^4^, ^5^

The crossmatch (XM) technique is used to determine donor‐recipient compatibility before blood transfusion. Based upon extensive clinical experience, dogs have no^6^, ^7^ or no clinically important naturally occurring alloantibodies, as anti‐DEA 7 antibodies that have been recently described.^8^, ^9^ Crossmatching before a first transfusion is not systematically required in dogs and is recommended when transfusion history is unknown, when a transfusion has been performed more than 4 days before or in a case of hemolytic reaction consecutive to a previous transfusion sensitization. Even when DEA 1‐matched blood is used for transfusion, some XM incompatibilities have been found 26 days after transfusion.^6^ Based on this observation, alloantibodies against blood groups other than DEA 1 are produced; therefore, XM must be performed after a first blood transfusion even when DEA 1 blood‐typing was performed. Xenotransfusion, that is, transfusion of blood from 1 species to another species, might be used only when an intraspecies donor is unavailable.^10^, ^11^ Although ethically questionable, this practice is occasionally used in cats with dog blood. Xenotransfusion from domestic dog to nondomesticated canids have only been reported twice.^12^, ^13^

*Canidae* is a family of carnivore mammals composed of 35 species; including wolf (*Canis lupus*) from whom domestic dog (*Canis lupus familiaris*) is a descendant. Based on phylogenetic studies, 4 clades have been discovered: “related to wolf,” “related to fox,” “South‐American canids,” and “2 species of *Urocyon* gender.”^14^ At this time, little is known about blood groups in nondomesticated canids and their incidence in incompatible transfusion.

The purpose of this prospective study was to assess the blood group DEA 1 for the first time in nondomesticated canids by using 2 technics of blood typing which are well known in domestic dogs^15^: immunochromatographic strip and flow cytometry. Thereafter, we also aimed to determine DEA 1+ and DEA 1− distribution depending on the species. The second objective was to investigate blood compatibilities between domestic dogs and nondomesticated canids and to determine if domestic canine blood could be used for xenotransfusion in nondomesticated canids when homologous blood is not available.

## 2 MATERIALS AND METHODS

### 2.1 Animals and blood sample collection

Blood samples were collected from nondomesticated canids living in French (n = 18) and Luxembourgish (n = 1) zoos, from whom veterinarians are members of the “Association Francophone des Vétérinaires de Parc Zoologique” (AFVPZ). One hundred and forty nondomesticated captive canids belonging to 13 species were included in this prospective study. According to phylogenetic studies,^14^ these canids were from 3 clades: “wolf” clade, “fox” clade, and “South America” clade. “Wolf” clade: wolf (*C lupus*) (n = 45), dhole (*Cuon alpinus*) (n = 16), wild dog (*Lycaon pictus*) (n = 13), dingo (*Canis lupus dingo*) (n = 3), and black‐backed jackal (*Canis mesomelas*) (n = 2). “Fox” clade: fennec fox (*Vulpes zerda*) (n = 17), red fox (*Vulpes vulpes*) (n = 12), arctic fox (*Vulpes lagopus*) (n = 5), raccoon dog (*Nyctereutes procyonoides*) (n = 4), corsac fox (*Vulpes corsac*) (n = 3), and bat‐eared fox (*Otocyon megalotis*) (n = 2). “South America” clade: bush dog (*Speothos venaticus*) (n = 10) and maned wolf (*Chrysocyon brachyurus*) (n = 8). Blood samples were opportunistically collected from those animals under anesthesia performed for care or physical exam for any reason.

For each specimen, approximately 3 mL of blood was collected into both ethylenediaminetetraacetic acid (EDTA) and dry tube. Dry tube was centrifuged to separate serum from red blood cells (RBC) and both tubes were sent to Dianov laboratories. Samples were preserved at 4°C and analyzed less than 1 week after sampling.^16^ Crossmatches between 2 animals were realized when the samples from these animals were treated in the same week.

Dog blood samples used for XM tests were collected during blood collection by the SIAMU (Intensive Care Unit, VetAgro Sup Veterinary campus) blood bank donors. Sixty‐three dogs were collected. These dogs were healthy and had not received any previous blood transfusion. This prospective study was approved by the Ethical Committee of VetAgro Sup (#1908).

### 2.2 Laboratory methods

For each canid, aliquots of EDTA‐anticoagulated whole blood samples were used for DEA 1 blood typing by immunochromatographic strip kit (Canine QuickTest DEA 1, Alvedia, Limonest, France) and the remaining blood was centrifuged at 1000*g* for 10 minutes to collect packed RBCs (pRBCs) for flow cytometric DEA 1 typing and stored at 4°C for XM testing within a week. Serum was separated on dry tubes and kept frozen at −20°C in a microtube between 1 week to a month for afterward XM testing.

#### 2.2.1 Dog erythrocyte antigen 1 typing

Dog erythrocyte antigen 1 typing was performed by 2 methods utilizing the same murine monoclonal anti‐DEA 1 antibody with a commercially available immunochromatographic strip kit and with a flow cytometric typing technique.

The immunochromatographic strip kit was used at bed side directly after blood collection by the veterinarians and once at the laboratory according to the manufacturer's instructions and as previously described,^15^ for results confirmation. The results were recorded as DEA 1 negative (no band) or DEA 1 positive (presence of a band).

For flow cytometric DEA 1 typing, 10 μL of pRBCs (<1‐week‐old) was washed 3 times with phosphate buffered saline (PBS). Then, 10 μL of a 10% washed RBC suspension in PBS was mixed with 100 μL of diluted murine monoclonal anti‐DEA 1 antibody (Alvedia, Limonest, France) and incubated at 37°C for 30 minutes. Thereafter, the RBC suspension was washed twice with PBS, and 100 μL of a 400‐fold diluted fluorescein isothiocyanate conjugated polyclonal goat anti‐mouse antibody solution (Abliance, Compiègne, France), was added to the RBC pellet. The suspension was mixed and incubated at 37°C for 30 minutes, washed again in PBS, and the pellet was resuspended in 500 μL of PBS prior to flow cytometric analysis using a FACSCalibur (Becton Dickinson & Co, Franklin Lakes, New Jersey). Data were collected and analyzed for 10 000 events through a gated region (CellQuest Pro software, Becton Dickinson & Co), and the mean fluorescence intensity (MFI) was obtained. The DEA 1 antigen RBCs' surface expression was designated as negative for a MFI < 10 and positive for any MFI ≥ 10.

#### 2.2.2 Gel column crossmatch without antiglobulin

Crossmatch tests were performed and interpreted according to the manufacturer's instructions (ID‐Cards NaCl, Enzyme Test and Cold Agglutinins, Bio‐Rad, DiaMed GmbH, Cressier, Switzerland) and as previously described.^6^ In a 3 mL polystyrene test tube, 50 μL of 1% donor pRBCs in low ionic strength solution (Bio‐Rad, DiaMed GmbH) were added at the top of the gel card column with 25 μL of recipient serum, briefly mixed, and incubated at 22°C for 10 minutes. After incubation, the gel column (GC) cards were centrifuged in a special GC centrifuge (ID Centrifuge 6S, Bio‐Rad, DiaMed GmbH) at 85*g* for 10 minutes, and the location of the migrated RBCs was recorded. In the absence of agglutination, the RBC passed through the gel to the bottom which was scored as “compatible,” whereas agglutination on the top of or within the gel was considered “incompatible.” Auto‐controls (using RBCs and plasma from same canid) were included for all XM tests performed.

Four varieties of XM assays have been performed in order to confirm that anti‐DEA 1 alloantibodies were the same in wild canids and in an immunized dog. For 1 sort, the plasma of a DEA 1− dog previously transfused with DEA 1+ blood, consequently possessing anti‐DEA 1 alloantibodies (sensitized dog plasma = SD plasma, thereafter)^17^ was tested with nondomesticated canids' pRBCs. One sort of XM assays was between nondomesticated canids' serum and dogs' pRBCs (major XM). The third one was between nondomesticated canids' pRBCs and dogs' plasma (minor XM). And the last one was between nondomesticated canids' pRBCs and other nondomesticated canids' serum.

### 2.3 Statistical analysis

Descriptive data are presented as average, range, and percentage. The blood typing test results were compared using McNemar test. The statistical analyses were performed using a commercially available statistical program (R, Saint‐Louis, Missouri), and a *P* ≤ .05 was considered significant.

## 3 RESULTS

### 3.1 DEA 1 typing results

For the immunochromatographic method, control bands were always present. The control band intensity was weak for some individuals: 9 fennec foxes, 9 red foxes, 3 corsac foxes, and 1 arctic fox. Results were identical regardless if performed by the veterinarian or by the laboratory staff.

The DEA 1 blood typing results were completely concordant between the flow cytometric and immunochromatographic strip typing techniques for 136 canids (Table 1). For 4 wolves (4/140, 2.9%), different results were obtained, depending on the blood typing technique.

**TABLE 1:** Nondomesticated canids DEA 1 blood typing results performed with immunochromatographic strip technique and flow cytometry technique

			Immunochromatographic strip, n (%)		Flow cytometry, n (%)	
Clade	Species	n	DEA 1+	DEA 1−	DEA 1+	DEA 1−
Wolf	Wolf (*Canis lupus*)	45	27 (60)	18 (40)	29 (64.4)	16 (35.6)
	Dhole (*Cuon alpinus*)	16	16 (100)	0 (0)	16 (100)	0 (0)
	Wild dog (*Lycaon pictus*)	13	13 (100)	0 (0)	13 (100)	0 (0)
	Dingo (*Canis lupus dingo*)	3	3 (100)	0 (0)	3 (100)	0 (0)
	Black‐backed jackal (*Canis mesomelas*)	2	2 (100)	0 (0)	2 (100)	0 (0)
	Subtotal	79	61 (77.2)	18 (22.8)	63 (79.7)	16 (20.3)
Fox	Fennec fox (*Vulpes zerda*)	17	17 (100)	0 (0)	17 (100)	0 (0)
	Red fox (*Vulpes vulpes*)	12	12 (100)	0 (0)	12 (100)	0 (0)
	Arctic fox (*Vulpes lagopus*)	5	3 (60)	2 (40)	3 (60)	2 (40)
	Raccoon dog (*Nyctereutes procyonoides*)	4	4 (100)	0 (0)	4 (100)	0 (0)
	Corsac fox (*Vulpes corsac*)	3	3 (100)	0 (0)	3 (100)	0 (0)
	Bat‐eared fox (*Otocyon megalotis*)	2	2 (100)	0 (0)	2 (100)	0 (0)
	Subtotal	43	41 (95.3)	2 (4.7)	41 (95.3)	2 (4.7)
South America	Bush dog (*Speothos venaticus*)	10	0 (0)	10 (100)	0 (0)	10 (100)
	Maned wolf (*Chrysocyon brachyurus*)	8	0 (0)	8 (100)	0 (0)	8 (100)
	Subtotal	18	0 (0)	18 (100)	0 (0)	18 (100)
Total		140	102 (72.9)	38 (27.1)	104 (74.3)	36 (25.7)

*Notes*: Results for 140 nondomesticated canids from 3 genetic clades: “wolf” clade, “fox” clade, and “South America” clade, representative of 13 species. n (%) indicates number (percentage) of nondomesticated canids.

**FIGURE 1:**
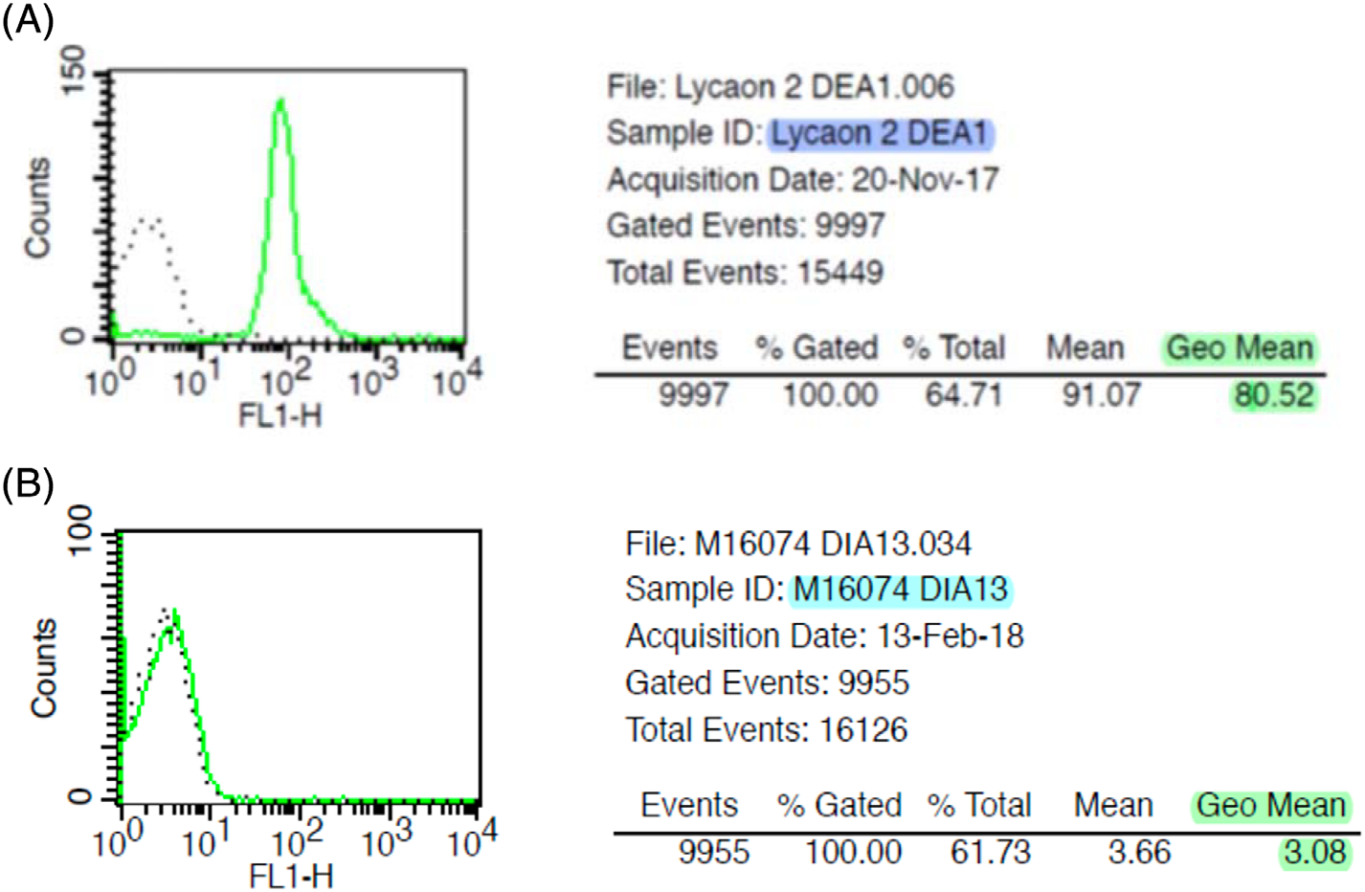
DEA 1 blood typing using flow cytometry technique in nondomesticated canids. A, A DEA 1+ wild dog, and B, a DEA 1− bush dog

Of 140 canids, 102 (72.9%) were DEA 1+ by immunochromatographic test and 104 (74.3%) were DEA 1+ (Figure 1A) by flow cytometric technique (Figure 1B). A McNemar test showed no significant difference (*P* = .62) between the 2 techniques.

All dholes (n = 16), wild dogs (n = 13), dingoes (n = 3), black‐backed jackals (n = 2), fennec foxes (n = 17), red foxes (n = 12), raccoon dogs (n = 4), corsac foxes (n = 3), and bat‐eared foxes (n = 2) were DEA 1+. In contrast, all bush dogs (n = 10) and maned wolves (n = 8) were DEA 1−. Wolf and arctic fox were the only species presenting both DEA 1+ and DEA 1− individuals.

### 3.2 Crossmatch results

#### 3.2.1 Autocontrol test results

There was no autoagglutination observed in any auto‐control tests when crossmatching serum and pRBCs from the same canid.

Four varieties of XM assays were performed:

- between nondomesticated canids' pRBCs and SD plasma,
- between nondomesticated canids' serum and dogs' pRBCs (major XM),
- between nondomesticated canids' pRBCs and dogs' plasma (minor XM), and
- between nondomesticated canids' pRBCs and other nondomesticated canids' serum.

#### 3.2.2 Crossmatch between nondomesticated canids RBCs and SD plasma^17^

Of the 140 nondomesticated canids packed RBCs tested, 112 (80%) incompatibilities were found. Incompatibilities were observed for 88 (79%) DEA 1+ nondomesticated canids and 24 (21%) DEA 1− nondomesticated canids. The XM was compatible for 28 (20%) canids, all wolves of which 16 were DEA 1+ (57%).

#### 3.2.3 Crossmatch between nondomesticated canids' serum and dogs' pRBCs: Major XM

One hundred and thirty nondomesticated canids were screened with a major XM against 1 or up to 8 donor dogs (median of 4 donor dogs): 1 donor dog (n = 14 canids), 2 donor dogs (n = 13 canids), 4 donor dogs (n = 51 canids), 5 donor dogs (n = 15 canids), 6 donor dogs (n = 31 canids), 7 donor dogs (n = 1 canid), and 8 donor dogs (n = 5 canids). When multiple donor dogs were used, both DEA 1+ and DEA 1− dogs were represented.

Ninety‐nine of 130 (76%) sera from nondomesticated canids were compatible with pRBCs from all donor dogs (Table 2). Fifty‐four of 69 (78%) individuals from the clade “wolf,” 35 of 43 (81%) individuals from the clade “fox,” and 10 of the 18 (56%) canids from the clade “South America” showed compatibilities on the major XM with all donor dogs (Table 2).

**TABLE 2:** RBC incompatibilities detected via gel column major crossmatch (between nondomesticated canids' serum and dogs' pRBCs) in different nondomesticated canids' clades

Number of crossmatch incompatibilities (n)	Number of donor dogs' RBCs tested (n)	Number of nondomesticated canids' sera			
		Clade “wolf” (n = 69)	Clade “fox” (n = 43)	Clade “South America” (n = 18)	All (n = 130)
0	1‐8	54	35	10	99
1	4	0	1	0	1
1	5	2	0	0	2
1	6	4	0	2	6
2	4	1	0	0	1
2	6	3	0	1	4
2	7	0	0	1	1
3	4	1	3	0	4
3	6	3	0	0	3
3	8	0	0	1	1
4	4	1	4	0	5
4	8	0	0	3	3
>0	1‐8	15	8	8	31

*Notes*: One hundred and thirty nondomesticated canids' sera were screened with 1 to 8 donor dogs' RBCs. 69 animals from clade “wolf”: 41 wolves, 12 dholes, 11 wild dogs, 3 dingoes, and 2 black‐backed jackals. 43 animals from clade “fox”: 17 fennec foxes, 12 red foxes, 5 arctic foxes, 4 raccoon dogs, 3 corsac foxes, and 2 bat‐eared foxes. Clade “South America”: 10 bush dogs and 8 maned wolves.

Abbreviations: n, number of nondomesticated canids or dogs; RBCs, red blood cells.

#### 3.2.4 Crossmatch between nondomesticated canids' pRBCs and dogs' plasma: Minor XM

Minor XMs were performed using pRBCs from 115 nondomesticated canids. Plasma from 1 to 7 donor dogs was used (median, 4 dogs): 1 donor (n = 8 canids), 2 donors (n = 3 canids), 3 donors (n = 18 canids), 4 donors (n = 63 canids), 5 donors (n = 13 canids), 6 donors (n = 9 canids), and 7 donors (n = 1 canid). When multiple donor dogs were used, DEA 1+ and DEA 1− dogs were represented.

**FIGURE 2:**
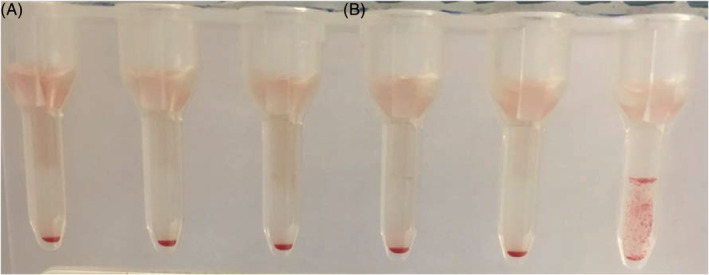
Crossmatching using neutral gel column technique. A, Five negative crossmatches (compatible) between 1 dhole pRBCs and 5 different donor dogs' plasma, B, positive crossmatch (incompatible) between 1 dhole pRBCs and 1 donor dog's plasma

Fifty‐nine of 115 (51%) pRBCs from nondomesticated canids were compatible with plasma from all donor dogs (Figure 2; Table 3). Forty‐nine of 62 (21%) individuals from the clade “wolf,” 5 of 35 (14%) canids from the clade “fox,” and 5 of 18 (28%) individuals from the clade “South America” showed compatibilities on the minor XM with all donor dogs (Table 3).

**TABLE 3:** RBC incompatibilities detected via gel column minor crossmatch (between nondomesticated canids' pRBCs and dogs' plasma) in different nondomesticated canids' clades

Number of crossmatch incompatibilities (n)	Number of donor dogs' plasma tested (n)	Number of nondomesticated canids' RBCs			
		Clade “wolf” (n = 62)	Clade “fox” (n = 35)	Clade “South America” (n = 18)	All (n = 115)
0	1‐7	49	5	5	59
1	3	4	0	1	5
1	4	2	2	2	6
2	3	2	6	0	8
2	4	2	6	3	11
2	5	2	0	0	2
2	6	1	0	0	1
3	3	0	2	0	2
3	4	0	7	7	14
3	5	0	6	0	6
4	4	0	1	0	1
> 0	1‐7	13	30	13	56

*Notes*: One hundred and fifteen nondomesticated canids' RBCs were screened with 1 to 7 donor dogs' plasma. 62 animals from clade “wolf”: 37 wolves, 14 dholes, 6 wild dogs, 3 dingoes, and 2 black‐backed jackals. 35 animals from clade “fox”: 11 fennec foxes, 11 red foxes, 4 arctic foxes, 4 raccoon dogs, 3 corsac foxes, and 2 bat‐eared foxes. 18 animals from clade “South America”: 10 bush dogs and 8 maned wolves.

Abbreviations: n, number of nondomesticated canids or dogs; RBCs, red blood cells.

#### 3.2.5 Crossmatch between nondomesticated canids' pRBCs and nondomesticated canids' serum

Firstly, major and minor XMs were performed between canids within the same species: 15 wolves (48 XMs), 11 dholes (54 XMs), 9 wild dogs (20 XMs), 3 dingoes (6 XMs), 2 black‐backed jackals (2 XMs), 17 fennec foxes (64 XMs), 9 red foxes (36 XMs), 4 raccoon dogs (12 XMs), 3 corsac foxes (6 XMs), 10 bush dogs (90 XMs), and 3 maned wolves (6 XMs). All the intraspecies XM test results were compatible.

Then, major and minor XM tests were performed between canids from different species. Within the same clade, XM tests results were compatible between 2 wild dogs and 1 dhole, 3 wolves and 2 dingoes, and 1 red fox and 1 arctic fox. However, some incompatibilities were observed among 3 wolves, 1 dhole, and 2 black‐backed jackals. For animals from different clades, incompatibilities were observed between 2 fennec foxes pRBCs and 2 wild dogs' serum. Crossmatches were compatible among 1 red fox, 1 dhole and 1 wolf, as well for 1 dhole and 2 fennec foxes.

## 4 DISCUSSION

Nondomesticated animals are less studied than domestic animals and a lot of specific features remain unknown, especially transfusion and blood compatibility. Few diagnostic tools have been developed for these species. The prospective study reported here assesses the blood group DEA 1 in nondomesticated canids by using 2 technics of blood typing which are well established in domestic dogs^15^: immunochromatographic strip and flow cytometry. The results indicate that nondomesticated canids do have DEA 1 RBC's surface antigens as well as domestic dogs. In veterinary medicine, quite a few blood typing kits used agglutination between patient RBC antigens and known antibody: agglutination card, immunochromatographic strip, automated blood‐typing, and gel test.^18^, ^19^, ^20^ Gel blood‐typing test has 100% specificity and sensibility.^18^ Unfortunately, this technique is no longer available, time consuming, and operator dependent. Other methods can be used in clinical settings and have been recently compared: immunochromatographic strip showed best performance.^18^, ^19^, ^20^ Flow cytometry is used only by research laboratories or when discording results appear. There is excellent correlation between flow cytometry and immunochromatographic strip techniques.^6^, ^15^ This study uses immunochromatographic strip method and laboratory flow cytometric technique, giving concordant results for the major part of nondomesticated canids (97.1%) and suggesting that immunochromatographic strip kit can be reliably used to type nondomesticated canids for DEA 1. Discordant typing results are obtained for 4 wolves (2.9%), MFI is close to 10 on flow cytometry analysis and could be considered as negative. Blood samples quality (hemolysis, autoagglutination) could affect flow cytometry results which analyzes only living cells. In those 4 animals, DEA 1 antigen expression on RBCs membrane remains inconclusive. The immunochromatographic strip incorporates a monoclonal antibody control band specific to canine glycophorin that guarantees the results interpretability in dogs. Occasionally, the intensity of reaction on control bands is weak for animals from “fox” clade, which is the most genetically distant clade from domestic dog. The antigen structure may have changed with genetic mutations.^14^

Several clinical studies reported DEA 1+ and DEA 1− prevalence depending on the region of the world and the breed of the dog. DEA 1+ dogs' prevalence ranges from 47 to 71%.^2^, ^3^, ^4^, ^21^ In the study reported here, the DEA 1+ and DEA 1− repartition is described for the first time in nondomesticated canids RBCs. The DEA 1+ prevalence is 72.9% (or 74.3%, depending on the technique). A majority of DEA 1− dogs have been reported in some breeds (Corso dog, Greyhound, Boxer, or German Shepherd).^3^, ^4^ In our study, some nondomesticated canids species, like maned wolf or bush dog, all tested animals are DEA 1−. Nevertheless, these species are underrepresented (18 of 140, 12.8%) and might explain the important prevalence of DEA 1+ animals. The DEA 1 mode of inheritance has been studied in dogs and is a multiallelic autosomal dominant blood system.^21^ In our study, animals are issued from 19 different zoos, although the DEA 1 prevalence could be biased since some were genetically linked. The heterogeneity of the species and the low number of individuals from each 1 yield to include more canids from other zoos to confirm this tendency or not.

Crossmatch tests are used to test blood compatibility between nondomesticated canids and domestic dogs. Gel column XM without antiglobulin technique is used; this method is adapted from the reference 1 in dogs.^1^ Autocontrol tests are all negative (no autoagglutination observed), so our results can be considered as reliable.

In this study, XM tests are performed between SD plasma^17^ with nondomesticated canids pRBCs, resulting in agglutination reactions in 80% of tested nondomesticated canids. Nondomesticated canids RBCs antigens seem to be well recognized by dogs' alloantibodies. Parts of incompatibilities are interpreted as caused by anti‐DEA 1 alloantibodies when DEA 1+ nondomesticated canids RBCs were tested (79%). Incompatibilities observed between DEA 1− nondomesticated canids RBCs (21%) and the SD are likely because of alloantibodies outside the DEA 1 system. Those alloantibodies could hypothetically react against blood type antigens that are present on RBCs membrane in both dogs and nondomesticated canids. One‐fifth of the nondomesticated canids RBCs, all wolves, are compatible with the SD plasma. These wolves were both DEA 1+ (57%) and DEA 1− (43%). The lack of agglutination reaction between DEA 1− nondomesticated canids RBCs and the SD can be easily understood. One can assume that the lack of agglutination reaction between DEA 1+ nondomesticated canids RBCs and the SD plasma could be the result of a poor alloantibody‐antigen recognition which could emanate from the antigen expression or a slight antigen structure change altering the antigenic conservation of the epitopes.^14^

In the study reported here, 3 quarters of nondomesticated canids show compatibilities with all dogs' RBCs tested, suggesting that most of nondomesticated canids lack naturally occurring alloantibodies against 1 or more canine blood groups. However, in parallel, tested dogs with no history of transfusion appear to have naturally occurring alloantibodies against half of tested nondomesticated canids tested.

Incompatibilities are recorded for either DEA 1+ or DEA 1− animals, therefore the DEA 1 blood group does not seem to be the only 1 involved in regards of these naturally occurring alloantibodies. In dogs, naturally occurring alloantibodies have only been recorded in vitro for the DEA 7 blood group.^9^ In matter of interspecies incompatibilities hypothesis, a previous study has performed XM between canine RBCs and feline plasma: no agglutination reactions were noted. Cats do not appear to have any naturally occurring alloantibodies against canine RBCs antigens.^10^ However, a more recent study has shown a high prevalence of naturally occurring antibodies in cats against DEAs and vice versa.^22^ In our case, alloantibodies detected could be against blood type antigens or might be against species specific antigens.

Crossmatch tests were performed without antiglobulin and potential reactions between nondomesticated canids' alloantibodies and canine antiglobulin were unknown to the authors' knowledge. Early data of the cross‐reactivity between nondomesticated canids' alloantibodies and commercially available canine antiglobulin was highlighted by the Ouchterlony technique (data not shown). Thus, for individuals possessing alloantibodies, potential cross‐reactions might occur during xenotransfusion however, the clinical impact is yet unknown. These early results indicate that major and minor XM are highly recommended before any transfusion of whole blood. Moreover, commercially available canine crossmatches based on a canine antiglobuline technology could be assessed for potential use on nondomesticated canids.

In our prospective study, intraspecies XM are all negatives suggesting a lack of naturally occurring alloantibodies, similar to dogs. When XM are performed between nondomesticated canids from different species, results are unpredictable.

Several studies had reported xenotransfusion of dog blood in cats.^10^, ^11^ The transfused canine RBCs had short lifespan and intravascular hemolysis occurred despite a clinically improvement within hours. In this study, the XM results indicated that a majority of domestic dogs' RBCs are compatible with nondomesticated canids' serum. Domestic dog could be a potential alternative donor because of the convenience of blood sampling, the great availability and it belonging to *Canidae* family. Clinical reactions and safety of xenotransfusion for nondomesticated canids using domestic dog blood is unknown, although 2 successful cases have been reported.^12^, ^13^ An island fox (*Urocyon littoralis clementae*) was bitten by a rattlesnake, causing anemia and a severe thrombocytopenia. A xenotransfusion using domestic dog whole blood was performed. No transfusion reactions were observed and the fox fully recovered.^12^ An arctic fox (*V lagopus*) presented an immune‐mediated hemolytic anemia. Xenotransfusion with pRBCs from a domestic dog was successfully used twice 24 hours apart. Pretransfusion major XM was compatible but 6 days posttransfusion, major XM was incompatible.^13^ Moreover, our results added to precedent reports^10^, ^13^ argue to the use of future xenotransfusion is risky because of alloantibodies induction leading to severe hemolytic reactions thus, when xenotransfusion is considered, XM is essential.

## 5 CONCLUSION

DEA 1 blood type exists in nondomesticated canids and DEA 1+ and DEA 1− distribution varies between species. Within the same species, studied animals do not possess any naturally occurring alloantibodies against blood type antigens, as in dogs. Blood typing of donor and recipient is recommended before any transfusion to prevent alloimmunization. Crossmatch could also be performed when transfusion history is unknown or previous transfusions have occurred. The majority of tested nondomesticated canids appear to have no naturally occurring alloantibodies against domestic dogs' RBCs yielding to consider xenotransfusion with dog's blood when same species donor is unavailable. However, 1 quarter of nondomesticated canids possess naturally occurring alloantibodies so, if xenotransfusion is considered, major and minor XM are essential.

## CONFLICT OF INTEREST DECLARATION

Maryline Guidetti was employed, and Isabelle Goy‐Thollot has been scientific advisor to Dianov. Reagents, commercial typing and crossmatch kits were provided for these studies by Alvedia. The design and execution of the study, data analysis, and writing of the manuscript have been done independently.

## OFF‐LABEL ANTIMICROBIAL DECLARATION

Authors declare no off‐label use of antimicrobials.

## INSTITUTIONAL ANIMAL CARE AND USE COMMITTEE (IACUC) OR OTHER APPROVAL DECLARATION

The study was approved by the IACUC of VetAgro Sup (#1908), and nondomesticated canid blood samples were obtained during anesthesia for a reason unrelated to this study.

## HUMAN ETHICS APPROVAL DECLARATION

Authors declare human ethics approval was not needed for this study.
